# Editorial on Special Issue “Marine Gels”

**DOI:** 10.3390/gels8030150

**Published:** 2022-02-28

**Authors:** Pedro Verdugo

**Affiliations:** Friday Harbor Laboratories, Department of Bioengineering, University of Washington, Friday Harbor, WA 98250, USA; verdugo@uw.edu

The ocean is a complex polymer solution. While Marine Sciences comprise a broad set of disciplines, polymer physics has remained largely absent in this front of inquiry. This Special Issue on marine gels is an attempt to alert marine scientists to the powerful predictive tools that physics offers to advance our understanding of the complex polymer dynamics taking place in the ocean. It is also an invitation to polymer physicists to participate in the urgent challenge of exploring the dynamics of marine biopolymers in the world’s oceans.

The cosmos, our last frontier, has received much attention, abundant funding, and important progress; however, the ocean, our first frontier—where life was borne and without which life would not exist on earth—remains a challenging field of underfunded riddles. Although explored for centuries, the utmost complexity of the ocean means that it remains one of the least understood subjects in the natural sciences. There is vast and excellent phenomenology at hand including detailed descriptions and correlations among an infinite number of variables; numerous mathematical models; taxonomy of marine living species from bacteria to whales; taxonomy of chemicals and more recent taxonomy of genes, present in seawater. However, the inner works of this gigantic reactor that keeps us alive remain largely a mystery. 

The cycling of carbon is the most critical thermodynamic process on our planet, and about half of it takes place in seawater. Understanding how CO_2_ is cycled in the ocean is a central issue for the survival of life on earth. While the overall map of carbon cycling is clear, the fundamentals of this process remain mostly unexplored. To remain alive, most living forms—humans in particular—combust organic matter, consume oxygen, and produce CO_2_. The reverse crucial cycling of this process results from photosynthesis, half of the global primary production of which is carried out by marine phytoplankton; carbon is fixed by photosynthesis that feeds higher trophic levels. The output of this gigantic photosynthetic reactor yields an annual mean value of ~50 Gt of carbon in the form of biopolymers. While the cell biology of phytoplankton remains obscure, we now know that these unicellulars function as secretory cells, storing biopolymers as condensed-phase polymer networks, and releasing them to seawater via the standard phase transition in exocytosis. The detailed mechanisms of what happens with the near 700 Pg of mostly polymeric-reduced organic material accumulated in seawater, remains hypothetical. What fraction reenters the cycle, consumed by marine biota, and why and what fraction join the recalcitrant organic stock—the most important disposal burial of organic carbon on the planet where discarded molecules remain buried for thousands of years—is still uncertain. Multifactorial modeling can be tweaked to account for multiple outcomes of marine mass transfer but, if untestable, their predictions often turn into formalized speculation. 

Despite polymers making up the bulk of organic stock present in the ocean, little of the powerful body of polymer physics, from Florey to Edwards, de Jeans, Dušek, and Tanaka’s, has been used to advance our understanding of marine polymer dynamics. Consequently, this Special Issue opens with a short tutorial on polymer networks theory. What follows is the assessment of the global distribution of marine biopolymers, and their chemical, polyelectrolyte, and hydrophobic features. Brief reviews of the application of polymer physics theory to explain the reversible association of biopolymers to form marine gels and the critical role of hydrophobic interactions in gel formation ensue. They provide interesting physical–chemical indications of why marine biopolymers either remain in the cycle or enter the largely irreversible recalcitrant burial. How bacteria gain access to metabolizable marine biopolymers is an equally important question, certainly not answered, but addressed here. The role of marine gels in bacterial nutrition, the ion-exchange of heavy metals, and the binding of pollutants is also addressed in this Special Issue. 

The deep, dark ocean stores most of the organic stock present in seawater; it presents some of the most intriguing questions in marine science, and has a special place in this set of reports. Finally, closing this set of news from the frontier is the recent and significant discovery that marine gels can be exported to the atmosphere, likely playing a significant role in cloud formation and climate change [1,2,3,4,5,6,7].

We, unfortunately, could to retrieve contributions from an important number of invited colleagues, particularly in marine microbiology and marine macrogel dynamics, whose collaboration would have expanded the scope of this issue. However, their work is thoroughly referenced to guide readers to their source.
